# Ancestral sequence alignment under optimal conditions

**DOI:** 10.1186/1471-2105-6-273

**Published:** 2005-11-17

**Authors:** Alexander K Hudek, Daniel G Brown

**Affiliations:** 1 School of Computer Science, University of Waterloo, 200 University Avenue West, Waterloo, Ontario, N2L 3G1, Canada

## Abstract

**Background:**

Multiple genome alignment is an important problem in bioinformatics. An important subproblem used by many multiple alignment approaches is that of aligning two multiple alignments. Many popular alignment algorithms for DNA use the sum-of-pairs heuristic, where the score of a multiple alignment is the sum of its induced pairwise alignment scores. However, the biological meaning of the sum-of-pairs of pairs heuristic is not obvious. Additionally, many algorithms based on the sum-of-pairs heuristic are complicated and slow, compared to pairwise alignment algorithms.

An alternative approach to aligning alignments is to first infer ancestral sequences for each alignment, and then align the two ancestral sequences. In addition to being fast, this method has a clear biological basis that takes into account the evolution implied by an underlying phylogenetic tree.

In this study we explore the accuracy of aligning alignments by ancestral sequence alignment. We examine the use of both maximum likelihood and parsimony to infer ancestral sequences. Additionally, we investigate the effect on accuracy of allowing ambiguity in our ancestral sequences.

**Results:**

We use synthetic sequence data that we generate by simulating evolution on a phylogenetic tree. We use two different types of phylogenetic trees: trees with a period of rapid growth followed by a period of slow growth, and trees with a period of slow growth followed by a period of rapid growth.

We examine the alignment accuracy of four ancestral sequence reconstruction and alignment methods: parsimony, maximum likelihood, ambiguous parsimony, and ambiguous maximum likelihood. Additionally, we compare against the alignment accuracy of two sum-of-pairs algorithms: ClustalW and the heuristic of Ma, Zhang, and Wang.

**Conclusion:**

We find that allowing ambiguity in ancestral sequences does not lead to better multiple alignments. Regardless of whether we use parsimony or maximum likelihood, the success of aligning ancestral sequences containing ambiguity is very sensitive to the choice of gap open cost. Surprisingly, we find that using maximum likelihood to infer ancestral sequences results in less accurate alignments than when using parsimony to infer ancestral sequences. Finally, we find that the sum-of-pairs methods produce better alignments than all of the ancestral alignment methods.

## Background

Multiple genome alignment is an important problem in bioinformatics. It is used in comparative studies to help find new genomic features such as genes and regulatory elements. Current multiple genome alignment programs [1,2] use progressive alignment [3], with a phylogenetic tree as a reference.

The primary operation of progressive alignment is the alignment of two multiple alignments. Most genome aligners have two main phases: anchoring and aligning between the anchors. Here, we focus on the algorithms used to align between anchors. Many popular alignment algorithms for DNA use the sum-of-pairs heuristic, where the score of a multiple alignment is the sum of the induced pairwise alignment scores. However, Just [4] has shown that finding the optimal alignment of two multiple alignments under the sum-of-pairs heuristic is NP-hard. Of course, since the problem is important, numerous heuristic algorithms [5-7] exist for alignment of alignment under sum of pairs.

The biological meaning of the sum-of-pairs of pairs heuristic is not obvious. Additionally, many heuristic algorithms are complicated and slow, compared to pairwise alignment algorithms [5-7]. An alternative approach to the strategy of aligning alignments under the sum-of-pairs heuristic is to first infer ancestral sequences for each alignment and then align the two ancestral sequences. In addition to being fast, this method has a clear biological basis that takes into account the evolution implied by the underlying tree.

Bray and Pachter use this approach to align alignments in MAVID [1]. MAVID uses maximum likelihood to infer ancestral sequences for alignments. In our previous work [8], we infer ancestral sequences for anchoring multiple alignments, but not for aligning alignments. Instead of maximum likelihood, we use parsimony to infer ancestral sequences, but we also allow these sequences to keep some ambiguity. The idea behind allowing ambiguity is to retain as much information about the underlying multiple alignment as possible. A natural question is whether aligning such ambiguous ancestral sequences leads to better alignments than aligning unambiguous ancestral sequences.

In this study, we explore this idea as well as other aspects of aligning alignments by ancestral sequence inference. We compare four ancestral alignment methods with two sum-of-pairs alignment algorithms. We infer ancestral sequences using parsimony and maximum likelihood, and study the effect of allowing ambiguity in these sequences. Since we are interested in the performance of these methods under optimal conditions, we use data generated by a very simple evolution simulation. For aligning full alignments with the sum-of-pairs heuristic, we use ClustalW [5] and a newer heuristic by Ma, Wang, and Zhang [6].

We find that alignment algorithms based on the sum-of-pairs heuristic are more accurate than all of our methods based on ancestral sequence alignment. However for alignment of inferred ancestral sequences, parsimony outperforms maximum likelihood in this application. Using maximum likelihood to infer ancestral sequences results in final alignment accuracies that are more unpredictable. Also, computing log-odds for ancestral sequences inferred with maximum likelihood is far more computationally intensive than computing log-odds scores for ancestral sequences inferred with parsimony. Finally, we find that allowing ancestral sequences to have ambiguity does not result in more accurate final alignments.

## Results

To determine whether using ambiguous symbols in ancestral sequences inference improves multiple alignment, we have performed experiments on simulated sequences. We propose five hypotheses, explain our experimental method, and finally discuss results and give conclusions.

### Our hypotheses

The first hypothesis is that by using ancestral sequences with ambiguity, we obtain more accurate multiple alignments. Ambiguous symbols may allow us to retain more information about the underlying multiple alignments, which may make it easier to identify matching positions. Combined with an appropriate log-odds scoring system, this extra information may allow for more accurate alignment of ancestral sequences, and by extension, for more accurate multiple alignments.

Our second hypothesis is that alignment of ancestral sequences is more sensitive to gap open costs than alignment of alignments using the sum-of-pairs heuristic. When aligning ancestral sequences, existing gaps in the underlying alignment are not considered when inserting a new gap, so the first position of a new gap always costs the gap open cost. Incorrect gap penalties may cause too many gaps to be inserted between ancestral sequences. During progressive alignment, errors at each step propagate leading to an incorrect final alignment. In contrast, when aligning alignments using the sum-of-pairs heuristic, the cost of adding a new gap depends on all underlying gaps as well as the gap open cost. An incorrect gap open cost affects the cost of gaps less and new gaps may still be correctly inserted based on the structure of the existing gaps. 

Our third hypothesis is that the function used to estimate the gap open cost during progressive alignment is important to alignment accuracy when aligning ancestral sequences. When aligning ancestral sequences, the frequencies of gaps in the ancestral sequences depends on the amount of mutation between the sequences. Therefore, it is important to modify the gap open cost based on the distance between the ancestral sequences being aligned.

Our fourth hypothesis is that we expect that aligning alignments using the sum-of-pairs heuristics gives more accurate multiple alignments than aligning inferred ancestral sequences. There are two reasons for this. First, as stated in hypothesis two, using an incorrect gap open cost affects the sum-of-pairs heuristic less than it affects the alignment of ancestral sequences. Since choosing the correct gap open cost can be difficult in practice, we expect that aligning alignments using the sum-of-pairs heuristic results in a more accurate final alignment because it is less sensitive to this parameter. Additionally, the ancestral sequences we infer are not completely accurate, which compounds the errors made in the process of progressive alignment. Thus, the much slower run times of algorithms based on the sum-of-pairs heuristic are acceptable.

Finally, we expect that the maximum likelihood methods result in better multiple alignments than the parsimony methods. Unlike parsimony, maximum likelihood uses the edge distances on the phylogenetic tree. Thus we expect maximum likelihood to better infer ancestral sequences.

### Experimental data

We use synthetic data in order to have correct alignments to test our methods against. Additionally, by generating our own data we ensure that the data is generated from the same model of evolution that is required for the alignment algorithm. Therefore, we consider the performance of the algorithms on this data to be the the best possible for algorithms of their type.

#### Phylogenetic trees

Despite our use of synthetic data, we want our data to mimic the basic properties of real biological sequence. Thus we generate random trees that resemble real trees, and assign mutation rates based on analysis of real sequences.

Specifically, we are interested in algorithm performance on two different types of random trees: trees with a period of heavy growth followed by a period of no growth, and trees with a period of light growth followed by a period of heavy growth. The first type of tree, which we refer to as early growth, is similar to the tree of placental mammals from Eizirik, Murphy, and O'Brien [9]. The second type of tree, which we refer to as late growth, is similar to the type of tree formed in a coalescent process of neutral mutation and speciation. We generate two sets of twenty phylogenetic trees, one set for each tree type. Each individual tree has eight taxa. We have chosen to limit our trees to trees with four taxa in each subtree of the root, both for ease of programming and to keep the time required to compute the log-odds scores for maximum likelihood reasonable.

We generate these in two steps. First, we generate one large tree of each type using a random birth-death process implemented in Phyl-O-Gen v1.2 [10]. To generate the early growth tree we start with a pure birth process with a birth rate of 0.35 events per million years. After we produce 100 lineages we switch to a birth-death process with both the birth and death rate set to 0.04 per million years. This episode lasts seven times as long as the first episode. We sample 66 lineages from the result, and this becomes our final tree, in Figure 3. We chose these values such that the tree resembles the tree of placental mammals both in topology and time scale. To generate the late growth tree we start with a pure birth process with a birth rate of 0.01 speciation events per million years. After eight speciation events, we switch to a birth rate of 0.20 speciation events per million years. We continue the process until we obtain 100 lineages. Again, we obtain a final tree by sampling 66 lineages from the result of the random process. This tree is shown in Figure 4. Here we choose values such that the tree has the same time scale and taxa as the early growth tree, but with a different topology.

**Figure 1:**
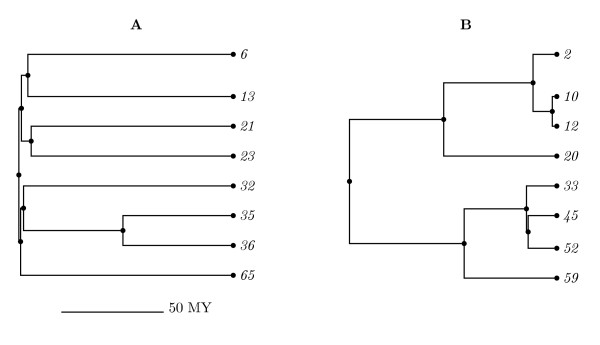
**Example random trees. **Example of a random tree with early growth (A) and a random tree with late growth (B).

**Figure 2:**
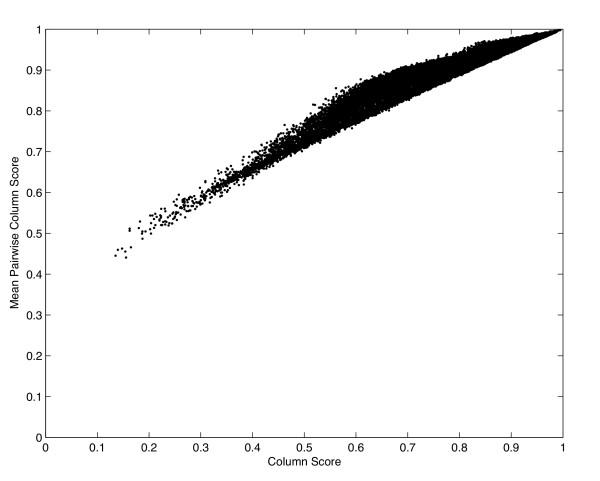
**Plot of column scores against mean pairwise column scores. **Plot of column accuracy versus mean pairwise column accuracy for alignments from all experiments. The column accuracy and the mean pairwise column accuracy have a roughly linear relationship.

**Figure 3:**
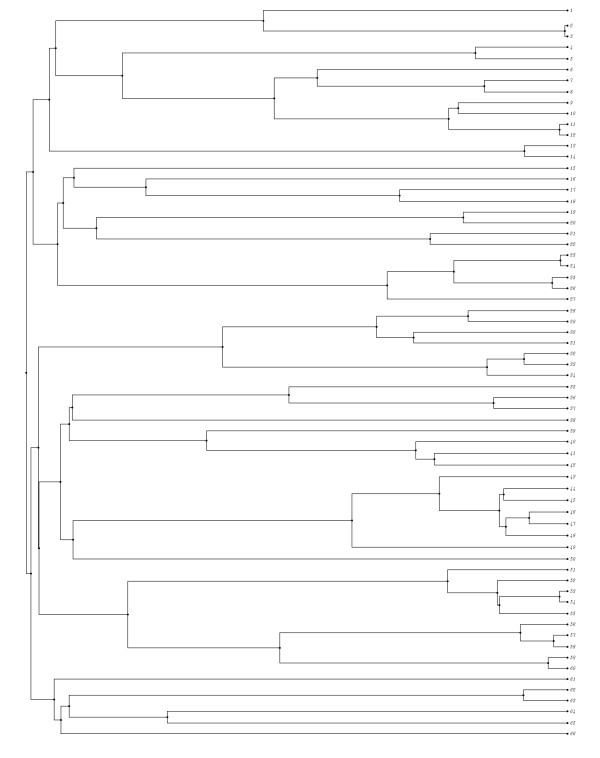
**Phylogenetic tree with early growth. **Phylogenetic tree with a period of rapid growth followed by a period of slow growth. This tree resembles the tree of placental mammals and the distance from the root to taxa is approximately 100 million years.

**Figure 4:**
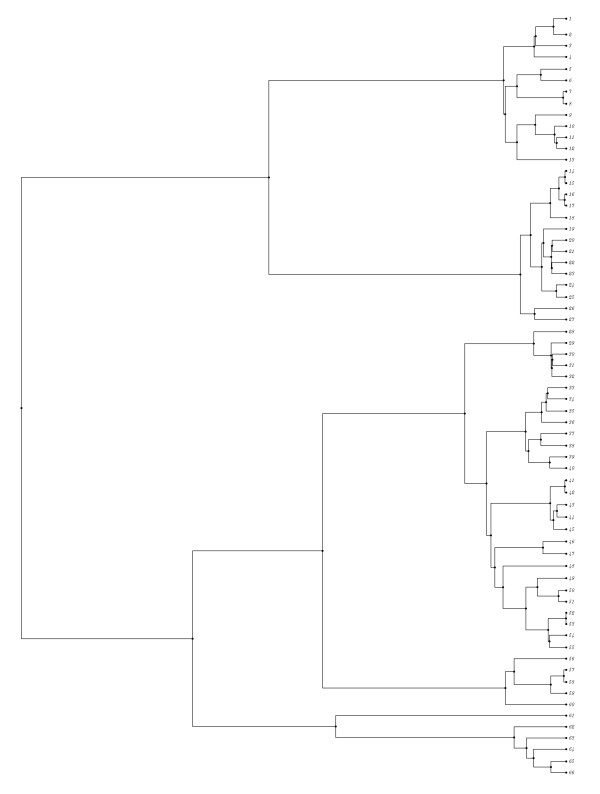
**Phylogenetic tree with late growth. **Phylogenetic tree with a period of slow growth followed by a period of rapid growth. The distance from root to taxa is approximately 100 million years.

From each final tree, using the method of Kearney, Munro, and Phillips [11], we randomly sample subtrees of eight taxa subject to the constraint that the amount of simulated time from the root to each taxon is between 90 to 110 million years. We then filter the resulting trees and only keep trees where the the left and right children have four descendants each. Examples of two of these trees are in Figure 1.

#### Random sequences

For each of the eight taxon trees in our two data sets, we generate twenty random sequence sets by simulating evolution over the tree. We use a program written by us, but similar to ROSE [12], to generate our sequences. For a given input tree, our program starts with an initial random sequence and mutates that sequence into new sequences down the tree. The program simulates Jukes/Cantor mutation events [13] as well as geometrically distributed insertion and deletion events with a mean length of 5.6. At the end, we have a set of sequences suitable as input to a multiple alignment program, and we have the original multiple alignment of the sequences.

Since we wanted our mutations, insertions, and deletions to be as close to real sequences as possible, we calibrated our simulator with parameters estimated through analysis of homologous human and baboon sequences. This gives us two sets of 400 random input sequences, one for each set of trees.

We chose the CFTR region in human and baboon for this parameter estimation, so that we could ensure that our alignment is mostly correct. We obtained human and baboon sequences with repeats masked out from the NISC Comparative Vertebrate Sequencing project [14]. We aligned a 10 kB region from each sequence and trimmed the ends of the alignments to obtain a final good alignment. From this final alignment, we measure the number of mutations as well as the length and number of gaps. Assuming that humans and baboons diverged approximately 25 million years ago (MYA), [15], and using the equation

Pr[mutation] = 3/4 (1 - *e^-rαt^*),     (1)

where *t *is the time in millions of years [13], we estimate the mutation rate *α *to be 7.1 × 10^-4 ^mutations per site per million years. We observe a rate of 4.1 × 10^-3 ^insertions or deletions per site. Assuming that insertions and deletions are equally likely and that we have only one possible insertion or deletion at a particular site, we find that Pr[insertion] = Pr[deletion] = 4.1 × 10^-5 ^events per site per million years. Additionally, we create another two sets of random input sequences using the same sets of trees, but with double the mutation rate on each tree branch.

### Experimental methods

We use the same insertion and deletion rates from our evolution simulator to compute the log-odds scores for the maximum likelihood methods. For a mean gap length of 5.6, we compute the optimal gap extension penalty to be 0.57 by standard methods [13]. When aligning alignments using the standard sum-of-pairs heuristic, any single gap cost is wrong for many pairs. Therefore, we should ideally use a different gap cost for each pair. However, as this increases the time complexity of Ma, Wang, and Zhang's algorithm, we instead use a single, unscaled gap cost for all pairwise alignments.

We test our first hypothesis by running all ancestral alignment methods and sum-of-pairs methods on all data sets using the optimal gap extension costs and a base gap open cost of 7. We use the *Expected *gap open cost estimation function since later tests show it to be better than the *Max *estimation function. To test our second hypothesis, we expand the previous test by exploring gap costs of 5 and 9. We test our third hypothesis by using two different scaling methods for each of the ancestral alignment methods. Our fourth and fifth hypothesis are also answered by the above three tests.

### Measuring success

We take the fraction of correct columns in an alignment to be the measure the alignment's accuracy. A correct column is one which contains the exact same nucleotides, from the same positions in the sequences, as a column in the correct alignment; a column with the same bases as a correct column, but from different positions, is incorrect. For a given data set and algorithm, we use the mean alignment accuracy of all 400 sequence data sets as a measure of the algorithm's accuracy.

We also considered an accuracy measure based on the pairwise alignments induced by the multiple alignment. In this, we compute the alignment accuracy of all induced pairwise alignments as in the previous paragraph and take the mean of these.

## Discussion

Before we discuss our results, we compare the two accuracy measures: column accuracy and mean pairwise column (MPC) accuracy. Tables 1 and 4 show that both measures have the same trends. Additionally, Figure 2 shows that the column accuracies and the MPC accuracies have a roughly linear relationship; the MPC accuracies are strictly higher than the column accuracies. Therefore, we do not give MPC accuracies for the experimental results in Tables 2 and 3. We now describe our experimental results with respect to our five hypotheses.

**Table 1:** Alignment accuracies using correct gap costs. Alignment accuracies using a gap open cost of 7 and the optimal gap extension cost of 0.57. P-values are computed using a paired Student's t-test.

Data Set	Measure	Parsimony	Ambiguous Parsimony	ML	Ambiguous ML	Ma et al.	ClustalW
Early Growth	Mean	88.43%	83.83%	87.86%	86.26%	91.74%	91.25%
	Std.	2.89%	5.94%	2.79%	4.27%	1.69%	2.28%
	P-value	1.6461 × 10^-65^		1.1992 × 10^-17^		N/A	
Early Growth Double Length	Mean	64.68%	57.24%	63.54%	59.89%	74.29%	72.35%
	Std.	5.23%	8.25%	4.99%	7.59%	4.62%	4.30%
	P-value	1.5922 × 10^-103^		1.9161 × 10^-21^		N/A	
Late Growth	Mean.	95.76%	96.01%	89.44%	87.52%	96.63%	96.79%
	Std	1.29%	1.28%	5.44%	7.03%	0.99%	1.10%
	P-value	7.7818 × 10^-10^		1.0404 × 10^-12^		N/A	
Late Growth Double Length	Mean	86.71%	88.12%	64.76%	55.67%	89.68%	89.54%
	Std.	2.71%	2.85%	7.94%	12.62%	2.42%	2.11%
	P-value	2.1699 × 10^-43^		2.5033 × 10^-50^		N/A	

**Table 2:** Alignment accuracies for differing gap open costs. Alignment accuracies for various gap open costs using a gap extension cost of 0.57.

Data Set	Gap Open Cost	Parsimony	Ambiguous Parsimony	ML	Ambiguous ML	Ma et al.	ClustalW
Early Growth	5	89.27%	88.41%	88.43%	88.09%	92.77%	90.48%
	7	88.43%	83.83%	87.86%	86.26%	91.74%	91.25%
	9	86.17%	76.04%	86.13%	82.58%	90.42%	91.48%
	Change	3.10%	12.37%	2.3%	5.51%	2.35%	1.00%
Early Growth Double Length	5	63.43%	63.32%	63.01%	63.05%	78.19%	69.63%
	7	64.68%	57.24%	63.54%	59.89%	74.29%	72.35%
	9	61.40%	46.72%	60.02%	52.33%	68.44%	73.59%
	Change	2.03%	16.6%	2.99%	10.72%	9.75%	3.96%
Late Growth	5	95.95%	96.35%	92.06%	91.70%	96.97%	96.50%
	7	95.76%	96.01%	89.44%	87.52%	96.63%	96.79%
	9	95.21%	95.39%	84.95%	80.71%	96.12%	96.91%
	Change	0.74%	0.96%	7.11%	10.99%	0.85%	0.41%
Late Growth Double Length	5	86.21%	88.91%	73.21%	68.18%	91.43%	88.17%
	7	86.71%	88.12%	64.76%	55.67%	89.68%	89.54%
	9	85.58%	86.16%	54.36%	43.02%	87.08%	90.15%
	Change	0.63%	2.75%	18.85%	25.16%	4.35%	1.98%

**Table 3:** Alignment accuracies using different gap open cost scaling functions. Alignment accuracies using two different gap cost scaling functions. The unsealed gap open cost is 7 and the unsealed gap extension cost is 1. The *Max *method scales gap open costs according to the maximum value in the scoring matrix. The *Expected *method scales gap open costs according to the expected score of a related symbol pair from the two ancestral sequences.

Data Set	Gap Scaling Method	Parsimony	Ambiguous Parsimony	ML	Ambiguous ML
Early Growth	Max	86.92%	85.07%	86.59%	86.13%
	Expected	89.95%	89.79%	89.17%	89.31%
Early Growth Double Length	Max	66.10%	64.51%	67.17%	66.10%
	Expected	71.12%	73.48%	72.02%	73.81%
Late Growth	Max	95.02%	95.21%	93.52%	92.31%
	Expected	96.04%	96.35%	95.03%	94.68%
Late Growth Double Length	Max	85.95%	86.39%	82.74%	75.98%
	Expected	88.38%	89.91%	87.17%	85.35%

**Table 4:** Mean pairwise alignment accuracies using correct gap costs. Mean pairwise alignment accuracies using a gap open cost of 7 and the optimal gap extension cost of 0.57. P-values are computed using a paired Student's t-test.

Data Set	Measure	Parsimony	Ambiguous Parsimony	ML	Ambiguous ML	Ma et al.	ClustalW
Early Growth	Mean	95.64%	92.61%	96.19%	95.41%	97.61%	97.48%
	Std.	1.43%	3.47%	1.08%	1.98%	0.48%	0.64%
	P-value	1.3830 × 10^-67^		9.6503 × 10^-17^		N/A	
Early Growth Double Length	Mean	84.33%	78.29%	84.30%	82.19%	91.20%	90.58%
	Std.	2.99%	5.50%	2.91%	4.95%	1.75%	1.51%
	P-value	9.1586 × 10^-114^		3.5959 × 10^-18^		N/A	
Late Growth	Mean	98.13%	98.26%	94.73%	93.74%	98.65%	98.67%
	Std.	0.62%	0.62%	3.14%	4.05%	0.39%	0.44%
	P-value	5.9142 × 10^-09^		1.1169 × 10^-10^		N/A	
Late Growth Double Length	Mean	93.63%	94.45%	80.79%	75.51%	95.50%	95.44%
	Std.	1.37%	1.47%	4.85%	7.91%	1.14%	0.95%
	P-value	2.1676 × 10^-44^		6.5523 × 10^-47^		N/A	

It is not clear that including ambiguity in ancestral sequences improves alignment. Looking at Tables 1 and 4, we see that on more than half the data sets, the ambiguous versions of parsimony and maximum likelihood have lower mean accuracies than their unambiguous counterparts. Additionally, ambiguous methods are less consistent in their scores, as evidenced by the larger standard deviations in the results for the ambiguous methods.

Table 2 shows mean alignment accuracies for the different data sets using a gap opening costs of 5, 7, and 9. For each data set and method, we measure the change in mean accuracy from a gap open cost of 5 to a gap open cost of 9. It is clear that the mean accuracy of the ambiguous methods changes far more than the mean accuracy of the unambiguous methods. Looking at the difference in mean accuracy of the ambiguous methods versus the unambiguous methods, we again see that the ambiguous methods are often lower than the unambiguous methods. Therefore, we conclude that the ambiguous methods are more sensitive to the gap open cost than the unambiguous methods.

Our experiment confirmed our hypothesis that the gap cost scaling function is very important to the resulting alignment accuracies. When changing from scaling based on the largest value in the ancestral sequence scoring matrix to the expected cost for related positions, we see a significant increase in alignment accuracy on all data sets and all methods. See Table 3 for results.

In Tables 1, 2, and 4, we see that both Ma, Wang, and Zhang's algorithm and ClustalW perform consistently better than the ancestral alignment methods. Also, Ma et al.'s algorithm performs better than ClustalW in most cases. However, the gap open cost affects Ma et al.'s algorithm and ClustalW differently. Depending on the choice of gap open cost, ClustalW may perform better than Ma et al.'s algorithm. Looking at all examined gap costs, Ma et al.'s algorithm achieves the highest alignment accuracy on each data set.

Surprisingly, the maximum likelihood methods performed worse than parsimony methods in the context of ancestral alignment. In Tables 1, 2, and 4, unambiguous maximum likelihood always performs worse than unambiguous parsimony. In some cases, such as on the Late Growth data set in Table 1, maximum likelihood performs drastically worse obtaining a mean alignment accuracy of 64.76% versus 86.71%. Looking at the mean alignment accuracy alone, it is not clear that ambiguous maximum likelihood is worse than ambiguous parsimony. However, on the late growth data set ambiguous parsimony does much worse than ambiguous parsimony. On other data sets, it performs similarly. Therefore, we conclude that ambiguous parsimony is more reliable than ambiguous maximum likelihood, if not more accurate.

## Conclusion

We have tested four ancestral alignment methods as well as two sum-of-pairs alignment methods on simulated data. The data mimics evolution on two types of evolutionary trees: trees with a period of rapid growth followed by a period of slow growth, and trees with a period of slow growth followed by a period of rapid growth. The four ancestral alignment methods we have tested are unambiguous parsimony, ambiguous parsimony, unambiguous maximum likelihood, and ambiguous maximum likelihood. The sum-of-pairs alignment methods we have tested are the ClustalW [5] algorithm and the algorithm of Ma, Wang, and Zhang [6].

We have found that, contrary to our hypotheses, allowing ambiguity in ancestral sequences does not lead to better alignments. When we use ambiguous ancestral sequences, we find that the multiple alignment is more sensitive to our choice in gap costs than to the form of ancestral sequence chosen. Reinforcing this conclusion, we find that the gap open cost scaling function is also extremely important to obtaining good scores when aligning ancestral sequences. Finally, to our surprise, using maximum likelihood to infer ancestral sequences resulting in less accurate alignments than using parsimony. The reason for this is that the maximum likelihood method is far more sensitive to the underlying data and therefore resulting in alignments accuracies that have a large amount of variation. Also, on the data set generated from the tree that has a small amount of growth followed by a large amount of growth, the maximum likelihood based methods did particularly poorly compared to the parsimony based methods.

Finally, both the sum-of-pairs approaches did better than all the ancestral alignment methods, as expected. Additionally, we found that Ma, Wang, and Zhang's algorithm [6] outperformed ClustalW [5] by a small amount.

## Methods

Our multiple alignment framework uses progressive alignment up a specified phylogenetic tree. At each internal node we perform an alignment of two multiple alignments. We test six different algorithms for aligning alignments: ClustalW, the recent algorithm of Ma, Wang, and Zhang, and four algorithms that align inferred ancestral sequences. Our four ancestral alignment algorithms explore the use of both parsimony and maximum likelihood to infer ancestral sequences, and also allow the use of both ambiguous and unambiguous ancestral sequences. We include ClustalW, as it is widely used in practice, and the algorithm of Ma, Wang, and Zhang, whose output more accurately approximates the optimal alignment under sum-of-pairs scoring.

In this section, we describe how we align two alignments using ancestral sequence inference, as well as our four ancestral sequence inference techniques and associated log-odds scoring frameworks.

### Alignment by ancestral sequence inference

Given two multiple alignments, we first infer an ancestral sequence for each alignment. Then, we remove gaps in the inferred ancestral sequences and align the them with the classic Needleman-Wunsch dynamic programming algorithm [16,17], under an appropriate log-odds scoring framework. Finally, we map the alignment of the two ancestral sequences to an alignment between the two alignments by inserting a column of gaps into each alignment for each gap inserted into the respective ancestral sequence. Given a length *n *alignment of *k *sequences, we can infer an ancestral sequence in time Θ(*kn*). To align two length *n *alignments, one with *k *sequences and with ℓ sequences, requires Θ((*k *+ ℓ)*n *+ *n*^2^) time; this contrasts with the much larger run time of *O *(*n*^2 ^(*k *+ ℓ)) required by Ma, Wang and Zhang's algorithm [6].

We explore four different methods of inferring and aligning ancestral sequences. First, we infer sequences using parsimony, finding both ambiguous and unambiguous ancestral sequences. Second, we infer sequences using maximum likelihood, again finding both ambiguous and unambiguous ancestral sequences. We call these methods parsimony, ambiguous parsimony, maximum likelihood, and ambiguous maximum likelihood. For each method, we also compute a log-odds scoring framework, which we use when we align two ancestral sequences.

To infer an ancestral sequence for a multiple alignment, we assume we have a correct edge weighted phylogenetic tree relating the sequences in the alignment. We work with a Jukes/Cantor 1-parameter evolution model [13], but our approach easily extends to more realistic models of evolution. We represent ambiguity in ancestral sequences using the 15-letter IUPAC alphabet. For example, if a position in an ancestral sequence is either an *A *or a *T*, we encode it as the IUPAC symbol *W*.

Let Γ represent the IUPAC alphabet and let Σ = {*A, C, G, T*} be the DNA alphabet. We represent a gap with the '-' character and define a third alphabet Σ_*G *_= Σ∪{-}. We use the symbol *b *to represent alphabet symbols from Σ or Σ_*G *_and *c *to represent symbols from Γ.

### Parsimony and ambiguous parsimony

The most parsimonious ancestral sequence is the sequence that requires the least number of mutations down the tree to obtain the sequences in the alignment. There can be multiple most parsimonious ancestral sequences for a given alignment. We take the union of these ancestral symbols at each position of the sequence, and thus obtain an ambiguous ancestral sequence. When we require an unambiguous ancestral sequence, we randomly choose a position at each point. We use an efficient algorithm by Fitch [18] to reconstruct both ambiguous and unambiguous ancestral sequences. We now briefly describe this algorithm.

Consider a single column of the alignment where the leaves of the tree are assigned the corresponding base, or gap from the column. Starting at the leaves of the tree we work upwards assigning an ambiguous symbol to each internal node. We do this with a *consensus *operation. If we consider each ambiguous symbol to be a set of possible bases, then the consensus of two symbols is the intersection of the sets, or the union if the intersection is empty. At a node *z *with children *x *and *y*, we assign *z *the consensus of the symbols at *x *and *y*. On completion, the symbol assigned to the root of the tree represents the set of most parsimonious ancestral bases for the column. Note that gaps are handled naturally: the consensus of a gap with any other symbol is the other symbol. Since each alignment column has at least one non-gap symbol, the reconstructed ancestral sequence contains no gaps.

To obtain an unambiguous ancestral base, we choose one of the bases represented by the root symbol uniformly at random. A consensus operation takes constant time, and so the above algorithm runs in *O*(*n*) time for a alignment column of *n *sequences.

#### Log-odds scoring framework

When aligning two ancestral sequences, we use the log-odds scoring framework from Brown and Hudek [8], explained in more detail in Hudek's thesis [19]. when aligning two ancestral sequences. We modify this framework slightly for unambiguous parsimony, and describe the framework and modification here.

Suppose we want to find an alignment at node *z*, with children *x *and *y *at which we have already computed multiple alignments. To do this, we align the inferred ancestral sequence at *x *to the inferred ancestral sequence at *y*. We score pairs of symbols from the two ancestral sequences using a log-odds framework based on the underlying phylogenetic tree.

We compute two probabilities: the probability Pr[*d*_1_,*d*_2_|related] of seeing the observed symbols *d*_1 _and *d*_2 _in ancestral sequence positions related by evolution according to the given tree, and Pr[*d*_1_, *d*_2_|unrelated], the probability of seeing observed symbols *d*_1 _and *d*_2 _in ancestral sequences positions unrelated by evolution. Here, *d*_1 _and *d*_2 _are either from the ambiguous or unambiguous alphabet depending on the type of ancestral sequence we use.

We start by computing *C*_*z*_(*c, b*), the probability that we see an ambiguous symbol *c *ε Γ in the ancestral sequence for node *z*, given that the true value of the ancestral sequence is the DNA base *b*.

**Theorem 1. ***For all nodes z and symbols c ε *Γ *and b *ε Σ, *the probability C*_*z*_(*c, b*) *can be computed in O*(*m*) *time where m is the number of descendants of node z and we treat *Γ *and *Σ as *constant in size*.

*Proof. *At the leaves, the ancestral symbol is the same as the associated alignment symbol. Therefore, we initially set *C*_*u*_(*c, b*) = 1 for all *c *and *b *where *c = b*, and *C*(*c, b*) = 0 for all others.

Let *T*_*v*_(*b*) be the event that the true value of the ancestral sequence at node *v *is the DNA base *b*. Let *C*_*c *_be the set of pairs of symbols (*c*_*x*_, *c*_*y*_) from Γ, whose consensus symbol is *c*. Consider a node *z *with children *x *and *y *for which we have already computed *C*_*v*_(*c, b*) for all values of *c *ε Γ and *b *ε Σ. We compute *C*_*z*_(*c*, *b*) using the equation

Cz(c,b)=∑  bx,by∈∑(cx,cy)∈ccDz,b(x,bx,cx)Dz,b(b,y,by,cy),     (2)
 MathType@MTEF@5@5@+=feaafiart1ev1aaatCvAUfKttLearuWrP9MDH5MBPbIqV92AaeXatLxBI9gBaebbnrfifHhDYfgasaacH8akY=wiFfYdH8Gipec8Eeeu0xXdbba9frFj0=OqFfea0dXdd9vqai=hGuQ8kuc9pgc9s8qqaq=dirpe0xb9q8qiLsFr0=vr0=vr0dc8meaabaqaciGacaGaaeqabaqabeGadaaakeaacqWGdbWqdaWgaaWcbaGaemOEaOhabeaakiabcIcaOiabdogaJjabcYcaSiabdkgaIjabcMcaPiabg2da9maaqafabaGaemiraq0aaSbaaSqaaiabdQha6jabcYcaSiabdkgaIbqabaGccqGGOaakcqWG4baEcqGGSaalcqWGIbGydaWgaaWcbaGaemiEaGhabeaakiabcYcaSiabdogaJnaaBaaaleaacqWG4baEaeqaaOGaeiykaKIaemiraq0aaSbaaSqaaiabdQha6jabcYcaSiabdkgaIbqabaGccqGGOaakcqWGIbGycqGGSaalcqWG5bqEcqGGSaalcqWGIbGydaWgaaWcbaGaemyEaKhabeaakiabcYcaSiabdogaJnaaBaaaleaacqWG5bqEaeqaaOGaeiykaKIaeiilaWcalqaabeqaaiaaykW7caaMc8UaemOyai2aaSbaaWqaaiabdIha4bqabaWccqGGSaalcqWGIbGydaWgaaadbaGaemyEaKhabeaaliabgIGiolabggHiLdqaaiabcIcaOiabdogaJnaaBaaameaacqWG4baEaeqaaSGaeiilaWIaem4yam2aaSbaaWqaaiabdMha5bqabaWccqGGPaqkcqGHiiIZcqWGJbWydaWgaaadbaGaem4yamgabeaaaaWcbeqdcqGHris5aOGaaCzcaiaaxMaadaqadaqaaiabikdaYaGaayjkaiaawMcaaaaa@7860@

where

*D*_*u*,*b *_(*v, b*_*v*_, *c*_*v*_) = Pr[*T*_*v*_(*b*_*v*_)|*T*_*u*_(*b*)]*C*_*v*_(*c*_*v*_, *b*_*v*_)     (3)

is the the probability of seeing *c*_*v *_at node *v *given the true value is *b*_*v*_, times the probability that the true value at node *v *is *b*_*v *_given that the true value at node *u *is *b*. We compute Pr[*T*_*v*_(*b*_*v*_)|*T*_*u*_(*b*)] using the length of the edge (*u, v*) and the probabilistic mutation model. We compute *C*_*v*_(*c*, *b*) for each node of the tree, and obtain *C*_*z*_(*c, b*) in *O*(*m*) time, where *m *is the number of leaves.     □

##### Ambiguous probabilities

When we use ambiguous ancestral sequences, the probability of seeing consensus letters *c*_1 _and *c*_2 _at a positions arising from a common ancestor is

Pr⁡[c1,c2|related]=∑bx,by,bz∈∑Pr⁡[Tx(bx),Ty(by)|Tz(bz)]Cx(c1,bx)Cy(c2,by).     (4)
 MathType@MTEF@5@5@+=feaafiart1ev1aaatCvAUfKttLearuWrP9MDH5MBPbIqV92AaeXatLxBI9gBaebbnrfifHhDYfgasaacH8akY=wiFfYdH8Gipec8Eeeu0xXdbba9frFj0=OqFfea0dXdd9vqai=hGuQ8kuc9pgc9s8qqaq=dirpe0xb9q8qiLsFr0=vr0=vr0dc8meaabaqaciGacaGaaeqabaqabeGadaaakeaacyGGqbaucqGGYbGCcqGGBbWwcqWGJbWydaWgaaWcbaGaeGymaedabeaakiabcYcaSiabdogaJnaaBaaaleaacqaIYaGmaeqaaOWaaqqaaeaacqqGYbGCcqqGLbqzcqqGSbaBcqqGHbqycqqG0baDcqqGLbqzcqqGKbazaiaawEa7aiabc2faDjabg2da9maaqafabaGagiiuaaLaeiOCaiNaei4waSLaemivaq1aaSbaaSqaaiabdIha4bqabaGccqGGOaakcqWGIbGydaWgaaWcbaGaemiEaGhabeaakiabcMcaPiabcYcaSiabdsfaunaaBaaaleaacqWG5bqEaeqaaOGaeiikaGIaemOyai2aaSbaaSqaaiabdMha5bqabaGccqGGPaqkdaabbaqaaiabdsfaunaaBaaaleaacqWG6bGEaeqaaOGaeiikaGIaemOyai2aaSbaaSqaaiabdQha6bqabaGccqGGPaqkaiaawEa7aiabc2faDjabdoeadnaaBaaaleaacqWG4baEaeqaaOGaeiikaGIaem4yam2aaSbaaSqaaiabigdaXaqabaGccqGGSaalcqWGIbGydaWgaaWcbaGaemiEaGhabeaakiabcMcaPiabdoeadnaaBaaaleaacqWG5bqEaeqaaOGaeiikaGIaem4yam2aaSbaaSqaaiabikdaYaqabaGccqGGSaalcqWGIbGydaWgaaWcbaGaemyEaKhabeaakiabcMcaPiabc6caUiaaxMaacaWLjaWaaeWaaeaacqaI0aanaiaawIcacaGLPaaaaSqaaiabdkgaInaaBaaameaacqWG4baEaeqaaSGaeiilaWIaemOyai2aaSbaaWqaaiabdMha5bqabaWccqGGSaalcqWGIbGydaWgaaadbaGaemOEaOhabeaaliabgIGiolabggHiLdqab0GaeyyeIuoaaaa@8C17@

That is, over all choices of the true value at *x*, *y*, and *z*, we compute the conditional probabilities of seeing consensus pair (*c*_1_, *c*_2_) at positions related by ancestry.

At positions unrelated by ancestry, we assume that the true value for the ancestral sequence is equally likely to be any of the four DNA bases, though this model can be made more complex to model more realistic sequences. Therefore, the probability of seeing consensus letters *c*_1 _and *c*_2 _is

Pr⁡[c1,c2|unrelated]=116∑bx∈ΣCx(c1,bx)∑by∈ΣCy(c2,by).     (5)
 MathType@MTEF@5@5@+=feaafiart1ev1aaatCvAUfKttLearuWrP9MDH5MBPbIqV92AaeXatLxBI9gBaebbnrfifHhDYfgasaacH8akY=wiFfYdH8Gipec8Eeeu0xXdbba9frFj0=OqFfea0dXdd9vqai=hGuQ8kuc9pgc9s8qqaq=dirpe0xb9q8qiLsFr0=vr0=vr0dc8meaabaqaciGacaGaaeqabaqabeGadaaakeaacyGGqbaucqGGYbGCdaWadaqaaiabdogaJnaaBaaaleaacqaIXaqmaeqaaOGaeiilaWIaem4yam2aaSbaaSqaaiabikdaYaqabaGcdaabbaqaaiabbwha1jabb6gaUjabbkhaYjabbwgaLjabbYgaSjabbggaHjabbsha0jabbwgaLjabbsgaKbGaay5bSdaacaGLBbGaayzxaaGaeyypa0ZaaSaaaeaacqaIXaqmaeaacqaIXaqmcqaI2aGnaaWaaabuaeaacqWGdbWqdaWgaaWcbaGaemiEaGhabeaakiabcIcaOiabdogaJnaaBaaaleaacqaIXaqmaeqaaOGaeiilaWIaemOyai2aaSbaaSqaaiabdIha4bqabaGccqGGPaqkaSqaaiabdkgaInaaBaaameaacqWG4baEaeqaaSGaeyicI4Saeu4OdmfabeqdcqGHris5aOWaaabuaeaacqWGdbWqdaWgaaWcbaGaemyEaKhabeaakiabcIcaOiabdogaJnaaBaaaleaacqaIYaGmaeqaaaqaaiabdkgaInaaBaaameaacqWG5bqEaeqaaSGaeyicI4Saeu4OdmfabeqdcqGHris5aOGaeiilaWIaemOyai2aaSbaaSqaaiabdMha5bqabaGccqGGPaqkcqGGUaGlcaWLjaGaaCzcamaabmaabaGaeGynaudacaGLOaGaayzkaaaaaa@7351@

Finally, the log-odds score for aligning consensus symbols *c*_1 _and *c*_2 _is

*S*(*c*_1_, *c*_2_) = log_2 _(Pr[*c*_1_, *c*_2_|related]/Pr[*c*_1_, *c*_2_|unrelated]),     (6)

in bits.

##### Unambiguous probabilities

If we use unambiguous ancestral sequences, the situation is similar. Let *V*(*b, c*) be the probability that we randomly choose base *b *from the set of bases represented by consensus symbol *c *∊ Γ. That is, if *c *is a symbol corresponding to a set of *k *symbols from Σ, and *b *is in this set, then *V*(*b*, *c*) = 1/*k*, otherwise it is zero. Then,

Pr[b1,b2|related]=∑bx,by,bz∈Σc1,c2∈ΓPr[Tx(bx),Ty(by)|Tz(bz)] Cx(c1,bx)V(b1,c1)Cy(c2,by)V(b2,c2)     (7)
 MathType@MTEF@5@5@+=feaafiart1ev1aaatCvAUfKttLearuWrP9MDH5MBPbIqV92AaeXatLxBI9gBaebbnrfifHhDYfgasaacH8akY=wiFfYdH8Gipec8Eeeu0dXdbba9frFj0=OqFfea0dXdd9vqai=hGuQ8kuc9pgc9s8qqaq=dirpe0xb9q8qiLsFr0=vr0=vr0dc8meaabaqaciGacaGaaeqabaqabeGadaaakeaacqqGqbaucqqGYbGCdaWadaqaaiabdkgaInaaBaaaleaacqaIXaqmaeqaaOGaeiilaWIaemOyai2aaSbaaSqaaiabikdaYaqabaGcdaabbaqaaiabbkhaYjabbwgaLjabbYgaSjabbggaHjabbsha0jabbwgaLjabbsgaKbGaay5bSdaacaGLBbGaayzxaaGaeyypa0ZaaabuaeaacqqGqbaucqqGYbGCdaWadaqaaiabdsfaunaaBaaaleaacqWG4baEaeqaaOGaeiikaGIaemOyai2aaSbaaSqaaiabdIha4bqabaGccqGGPaqkcqGGSaalcqWGubavdaWgaaWcbaGaemyEaKhabeaakiabcIcaOiabdkgaInaaBaaaleaacqWG5bqEaeqaaOGaeiykaKYaaqqaaeaacqWGubavdaWgaaWcbaGaemOEaOhabeaakiabcIcaOiabdkgaInaaBaaaleaacqWG6bGEaeqaaOGaeiykaKcacaGLhWoaaiaawUfacaGLDbaaaSqaauaabeqaceaaaeaacqWGIbGydaWgaaadbaGaemiEaGhabeaaliabcYcaSiabdkgaInaaBaaameaacqWG5bqEaeqaaSGaeiilaWIaemOyai2aaSbaaWqaaiabdQha6bqabaWccqGHiiIZcqqHJoWuaeaacqWGJbWydaWgaaadbaGaeGymaedabeaaliabcYcaSiabdogaJnaaBaaameaacqaIYaGmaeqaaSGaeyicI4Saeu4KdCeaaaqab0GaeyyeIuoakiaaykW7cqWGdbWqdaWgaaWcbaGaemiEaGhabeaakiabcIcaOiabdogaJnaaBaaaleaacqaIXaqmaeqaaOGaeiilaWIaemOyai2aaSbaaSqaaiabdIha4bqabaGccqGGPaqkcqWGwbGvcqGGOaakcqWGIbGydaWgaaWcbaGaeGymaedabeaakiabcYcaSiabdogaJnaaBaaaleaacqaIXaqmaeqaaOGaeiykaKIaem4qam0aaSbaaSqaaiabdMha5bqabaGccqGGOaakcqWGJbWydaWgaaWcbaGaeGOmaidabeaakiabcYcaSiabdkgaInaaBaaaleaacqWG5bqEaeqaaOGaeiykaKIaemOvayLaeiikaGIaemOyai2aaSbaaSqaaiabikdaYaqabaGccqGGSaalcqWGJbWydaWgaaWcbaGaeGOmaidabeaakiabcMcaPiaaxMaacaWLjaWaaeWaaeaacqaI3aWnaiaawIcacaGLPaaaaaa@A5AC@

is the probability that we see DNA base *b*_1 _at *x *and DNA base *b*_2 _at *y *given that we are looking at positions arising from a common ancestor.

At positions unrelated by ancestry, we assume that the true value for the ancestral sequence is equally likely to be any of the four DNA bases. Therefore, the probability of seeing DNA base *b*_1 _and *b*_2 _is

Pr[b1,b2|unrelated]=116∑c1∈Γbx∈ΣCx(c1,bx)V(b1,c1)∑c2∈Γby∈ΣCy(c2,by)V(b2,c2).     (8)
 MathType@MTEF@5@5@+=feaafiart1ev1aaatCvAUfKttLearuWrP9MDH5MBPbIqV92AaeXatLxBI9gBaebbnrfifHhDYfgasaacH8akY=wiFfYdH8Gipec8Eeeu0xXdbba9frFj0=OqFfea0dXdd9vqai=hGuQ8kuc9pgc9s8qqaq=dirpe0xb9q8qiLsFr0=vr0=vr0dc8meaabaqaciGacaGaaeqabaqabeGadaaakeaacqqGqbaucqqGYbGCdaWadaqaaiabdkgaInaaBaaaleaacqaIXaqmaeqaaOGaeiilaWIaemOyai2aaSbaaSqaaiabikdaYaqabaGcdaabbaqaaiabbwha1jabb6gaUjabbkhaYjabbwgaLjabbYgaSjabbggaHjabbsha0jabbwgaLjabbsgaKbGaay5bSdaacaGLBbGaayzxaaGaeyypa0ZaaSaaaeaacqaIXaqmaeaacqaIXaqmcqaI2aGnaaWaaabuaeaacqWGdbWqdaWgaaWcbaGaemiEaGhabeaakiabcIcaOiabdogaJnaaBaaaleaacqaIXaqmaeqaaOGaeiilaWIaemOyai2aaSbaaSqaaiabdIha4bqabaGccqGGPaqkcqWGwbGvcqGGOaakcqWGIbGydaWgaaWcbaGaeGymaedabeaakiabcYcaSiabdogaJnaaBaaaleaacqaIXaqmaeqaaOGaeiykaKcalqaabeqaaiabdogaJnaaBaaameaacqaIXaqmaeqaaSGaeyicI4Saeu4KdCeabaGaemOyai2aaSbaaWqaaiabdIha4bqabaWccqGHiiIZcqqHJoWuaaqab0GaeyyeIuoakmaaqafabaGaem4qam0aaSbaaSqaaiabdMha5bqabaGccqGGOaakcqWGJbWydaWgaaWcbaGaeGOmaidabeaakiabcYcaSiabdkgaInaaBaaaleaacqWG5bqEaeqaaOGaeiykaKIaemOvayLaeiikaGIaemOyai2aaSbaaSqaaiabikdaYaqabaGccqGGSaalcqWGJbWydaWgaaWcbaGaeGOmaidabeaakiabcMcaPiabc6caUiaaxMaacaWLjaWaaeWaaeaacqaI4aaoaiaawIcacaGLPaaaaSabaeqabaGaem4yam2aaSbaaWqaaiabikdaYaqabaWccqGHiiIZcqqHtoWraeaacqWGIbGydaWgaaadbaGaemyEaKhabeaaliabgIGiolabfo6atbaabeqdcqGHris5aaaa@8F92@

The log-odds score for DNA symbols *b*_1 _and *b*_2 _is

*S*(*b*_1_, *b*_2_) = log_2 _(Pr[*b*_1_, *b*_2_|related]/Pr[*b*_1_, *b*_2_|unrelated]),     (9)

in bits.

### Maximum likelihood

For a given alignment column, we compute the most likely ancestral DNA base as in Felsenstein [20], but modified to consider gap characters in the column. We model the gap symbol as other symbols in the alphabet, but use special probabilities when considering changes to and from gap characters. Specifically, we use an insertion/deletion probability whenever we consider a mutation from a non-gap symbol to a gap symbol, or a gap symbol to a non-gap symbol. The probability of going from a gap symbol to a gap symbol is one minus the probability of an insertion.

Upon completion of the basic inference algorithm, we have a vector at the root that gives, for each position and each DNA base, the likelihood of that base. Assuming independence, the likelihood of a given ancestral sequence is the product of these. We obtain an unambiguous ancestral sequence from this by taking the base with maximum likelihood, randomly choosing between ties. To obtain an ambiguous maximum likelihood, which in effect is an approximation of the posterior Bayesian distribution of the ancestral symbol at that site, where we assume a uniform prior distribution over all alphabet symbols, we map the vector to an IUPAC symbol as follows. For each IUPAC symbol, we define a vector over the DNA alphabet where we have a one for each DNA symbol described by the IUPAC symbol, and a zero for all other DNA symbols. For example, the IUPAC symbol *W *represents an *A *or a T. We define associated vector (1,1,0,0), scaled to a probability distribution, where the numbers in the vector refer to *A,T,C*, and *G*, in that order. We then map the likelihood vector, also scaled to a probability vector (which, again, corresponds to computing the posterior probabilities, assuming a flat prior on all four DNA bases though this assumption can easily be removed), to an IUPAC symbol, choosing the IUPAC symbol with associated vector that has the closest euclidean distance to the likelihood vector scaled to a probability distribution.

#### Log-odds scoring framework

We desire a log-odds scoring framework similar to that developed for parsimonious ancestral sequences. While an appropriate scoring matrix can be obtained by sampling, we want to eliminate any sampling error from our study and so choose to compute the log-odds scoring framework directly.

Assume we are at node *z *with children *x *and *y *in a tree rooted at node *r*. First, consider the sub tree rooted at *x*. Let Lx,d
 MathType@MTEF@5@5@+=feaafiart1ev1aaatCvAUfKttLearuWrP9MDH5MBPbIqV92AaeXatLxBI9gBamXvP5wqSXMqHnxAJn0BKvguHDwzZbqegm0B1jxALjhiov2DaebbnrfifHhDYfgasaacH8akY=wiFfYdH8Gipec8Eeeu0xXdbba9frFj0=OqFfea0dXdd9vqai=hGuQ8kuc9pgc9s8qqaq=dirpe0xb9q8qiLsFr0=vr0=vr0dc8meaabaqaciaacaGaaeqabaWaaeGaeaaakeaaimaacaWFmbWaaSbaaSqaaiabdIha4jabcYcaSiabdsgaKbqabaaaaa@3D3D@ be the set of all assignments of symbols from Σ_*G *_to the leaves of the sub tree rooted at *x *such that maximum likelihood infers symbol *d *at *x*.

Let *O*_*x*_(*a*) be the probability that a particular assignment *a *of bases to the leaves of sub tree *x *occurs by evolution. We compute the probability that the true DNA base at node *x *is *b *by considering the probability all possible bases for the root of the tree, and simulating evolution down to *b*.

In the following, as we compute scores for unambiguous ancestral sequences, *d*_1 _and *d*_2 _refer to symbols from Σ_*G*_. To compute scores for ambiguous ancestral sequences, *d*_1 _and *d*_2 _refer to symbols from Γ. We compute Pr[*d*_1_,*d*_2_|related], the probability of seeing symbols *d*_1 _and *d*_2 _in positions related by a common ancestor, as

Pr[d1,d2|related]=∑ax∈Lx,d1ay∈Ly,d2   bz∈ΣGPr[Ox(ax)|Tz(bz)]Pr⁡[Oy(ay)|Tz(bz)]Pr[Tz(bz)],     (10)
 MathType@MTEF@5@5@+=feaafiart1ev1aaatCvAUfKttLearuWrP9MDH5MBPbIqV92AaeXatLxBI9gBamXvP5wqSXMqHnxAJn0BKvguHDwzZbqegm0B1jxALjhiov2DaebbnrfifHhDYfgasaacH8akY=wiFfYdH8Gipec8Eeeu0xXdbba9frFj0=OqFfea0dXdd9vqai=hGuQ8kuc9pgc9s8qqaq=dirpe0xb9q8qiLsFr0=vr0=vr0dc8meaabaqaciaacaGaaeqabaWaaeGaeaaakeaacqqGqbaucqqGYbGCcqqGBbWwcqWGKbazdaWgaaWcbaGaeGymaedabeaakiabcYcaSiabdsgaKnaaBaaaleaacqaIYaGmaeqaaOGaeiiFaWNaeeOCaiNaeeyzauMaeeiBaWMaeeyyaeMaeeiDaqNaeeyzauMaeeizaqMaeeyxa0Laeyypa0ZaaabuaeaacqqGqbaucqqGYbGCcqqGBbWwcqWGpbWtdaWgaaWcbaGaemiEaGhabeaakiabcIcaOiabdggaHnaaBaaaleaacqWG4baEaeqaaOGaeiykaKIaeiiFaWNaemivaq1aaSbaaSqaaiabdQha6bqabaGccqGGOaakcqWGIbGydaWgaaWcbaGaemOEaOhabeaakiabcMcaPiabc2faDjGbccfaqjabckhaYjabcUfaBjabd+eapnaaBaaaleaacqWG5bqEaeqaaOGaeiikaGIaemyyae2aaSbaaSqaaiabdMha5bqabaGccqGGPaqkcqGG8baFcqWGubavdaWgaaWcbaGaemOEaOhabeaakiabcIcaOiabdkgaInaaBaaaleaacqWG6bGEaeqaaOGaeiykaKIaeiyxa0LaeeiuaaLaeeOCaiNaee4waSLaemivaq1aaSbaaSqaaiabdQha6bqabaGccqGGOaakcqWGIbGydaWgaaWcbaGaemOEaOhabeaakiabcMcaPiabc2faDjabcYcaSiaaxMaacaWLjaWaaeWaaeaacqaIXaqmcqaIWaamaiaawIcacaGLPaaaaSabaeqabaGaemyyae2aaSbaaWqaaiabdIha4bqabaWccqGHiiIZimaacaWFmbWaaSbaaWqaaiabdIha4bqabaWccqGGSaalcqWGKbazdaWgaaadbaGaeGymaedabeaaaSqaaiabdggaHnaaBaaameaacqWG5bqEaeqaaSGaeyicI4Saa8htamaaBaaameaacqWG5bqEaeqaaSGaeiilaWIaemizaq2aaSbaaWqaaiabikdaYaqabaaaleaacaaMc8UaaGPaVlaaykW7cqWGIbGydaWgaaadbaGaemOEaOhabeaaliabgIGiolabfo6atnaaBaaameaacqWGhbWraeqaaaaaleqaniabggHiLdaaaa@AFD9@

where we compute the probability that the true value at node *z *is *b *as

Pr[Tz(b)]=∑br∈ΣGPr[Tz(b)|Tr(br)] Pr[Tr(br)].     (11)
 MathType@MTEF@5@5@+=feaafiart1ev1aaatCvAUfKttLearuWrP9MDH5MBPbIqV92AaeXatLxBI9gBaebbnrfifHhDYfgasaacH8akY=wiFfYdH8Gipec8Eeeu0xXdbba9frFj0=OqFfea0dXdd9vqai=hGuQ8kuc9pgc9s8qqaq=dirpe0xb9q8qiLsFr0=vr0=vr0dc8meaabaqaciGacaGaaeqabaqabeGadaaakeaacqqGqbaucqqGYbGCdaWadaqaaiabdsfaunaaBaaaleaacqWG6bGEaeqaaOGaeiikaGIaemOyaiMaeiykaKcacaGLBbGaayzxaaGaeyypa0ZaaabuaeaacqqGqbaucqqGYbGCdaWadaqaaiabdsfaunaaBaaaleaacqWG6bGEaeqaaOGaeiikaGIaemOyaiMaeiykaKYaaqqaaeaacqWGubavdaWgaaWcbaGaemOCaihabeaakiabcIcaOiabdkgaInaaBaaaleaacqWGYbGCaeqaaOGaeiykaKcacaGLhWoaaiaawUfacaGLDbaaaSqaaiabdkgaInaaBaaameaacqWGYbGCaeqaaSGaeyicI4Saeu4Odm1aaSbaaWqaaiabdEeahbqabaaaleqaniabggHiLdGccqqGGaaicqqGqbaucqqGYbGCdaWadaqaaiabdsfaunaaBaaaleaacqWGYbGCaeqaaOGaeiikaGIaemOyai2aaSbaaSqaaiabdkhaYbqabaGccqGGPaqkaiaawUfacaGLDbaacqGGUaGlcaWLjaGaaCzcamaabmaabaGaeGymaeJaeGymaedacaGLOaGaayzkaaaaaa@6754@

That is, for each possible true value at root *r*, we compute the probability that the value mutates to *b *on the path from *r *to *z*.

We now compute Pr[*d*_1_,*d*_2_|unrelated], the probability that we see symbols *d*_1 _and *d*_2 _in positions unrelated by a common ancestor. First, let *ML*_*x*_(*d*) be the event that maximum likelihood infers symbol *d *at node *x*. We compute *ML*_*x*_(*d*) as

Pr[MLx(d)]=∑a∈Lx,d  b∈ΣGPr[Ox(a)|Tx(b)]Pr[Tx(b)]     (12)
 MathType@MTEF@5@5@+=feaafiart1ev1aaatCvAUfKttLearuWrP9MDH5MBPbIqV92AaeXatLxBI9gBamXvP5wqSXMqHnxAJn0BKvguHDwzZbqegm0B1jxALjhiov2DaebbnrfifHhDYfgasaacH8akY=wiFfYdH8Gipec8Eeeu0xXdbba9frFj0=OqFfea0dXdd9vqai=hGuQ8kuc9pgc9s8qqaq=dirpe0xb9q8qiLsFr0=vr0=vr0dc8meaabaqaciaacaGaaeqabaWaaeGaeaaakeaacqqGqbaucqqGYbGCcqGGBbWwcqWGnbqtcqWGmbatdaWgaaWcbaGaemiEaGhabeaakiabcIcaOiabdsgaKjabcMcaPiabc2faDjabg2da9maaqafabaGaeeiuaaLaeeOCaiNaei4waSLaem4ta80aaSbaaSqaaiabdIha4bqabaGccqGGOaakcqWGHbqycqGGPaqkcqGG8baFcqWGubavdaWgaaWcbaGaemiEaGhabeaakiabcIcaOiabdkgaIjabcMcaPiabc2faDjabbcfaqjabbkhaYjabbUfaBjabdsfaunaaBaaaleaacqWG4baEaeqaaOGaeiikaGIaemOyaiMaeiykaKIaeiyxa0LaaCzcaiaaxMaadaqadaqaaiabigdaXiabikdaYaGaayjkaiaawMcaaaWceaqabeaacqWGHbqycqGHiiIZimaacaWFmbWaaSbaaWqaaiabdIha4jabcYcaSiabdsgaKbqabaaaleaacaaMc8UaaGPaVlabdkgaIjabgIGiolabfo6atnaaBaaameaacqWGhbWraeqaaaaaleqaniabggHiLdaaaa@79ED@

where we compute Pr[*T*_*x*_(*b*)] in the same way as Pr[*T*_*z*_(*b*)] previously. We compute Pr[*d*_1_, *d*_2_|unrelated] as

Pr[*d*_1_, *d*_2_|unrelated] = Pr[*ML*_*x*_(*d*_1_)] Pr[*ML*_*y*_(*d*_2_)].     (13)

Finally, the log-odds score of symbols *d*_1 _and *d*_1 _is

*S*(*d*_1_, *d*_2_) = log_2 _(Pr[*d*_1_, *d*_2_|related]/Pr[*d*_1_, *d*_2_|unrelated]).     (14)

To compute the above scores, we must examine every possible assignment of bases to leaves for both the left and right children of the root. For a particular tree, let *k *be the maximum of the number of taxa below the left child of the root and the right child of the root. We require time *O*(|Σ_*G*_|^*k*^) to compute the log-odds scores for this tree.

### Gap open cost scaling

When aligning alignments with ancestral sequences, gap open costs play a major role. In a tree with differing edge lengths, the gap open cost should also be able to vary. Since existing gaps are not considered when inserting a new gap in ancestral alignments, the gap open cost has a large influence over the quality of the resulting alignment. It is therefore important that we estimate an appropriate gap open cost for each node of the tree.

Consider creating alignment for node *z *with children *x *and *y*. We scale the gap open cost according to the distance between the nodes *x *and *y*. We test two slightly different scaling functions. The first function multiplies the gap open cost by the largest score value in the log-odds scoring matrix for node *z*. We call this the *Max *method. The second function computes the expected score of two symbols from unrelated positions and uses this value to scale the gap open cost. We call this the *Expected *method.

## Authors' contributions

Both AH and DB developed the fundamental ideas and hypotheses and the mathematical framework for deriving the alignment scores. AH developed and ran the experiments and implemented the alignment algorithms.
